# Structural basis of unique ligand specificity of KAI2-like protein from parasitic weed *Striga hermonthica*

**DOI:** 10.1038/srep31386

**Published:** 2016-08-10

**Authors:** Yuqun Xu, Takuya Miyakawa, Hidemitsu Nakamura, Akira Nakamura, Yusaku Imamura, Tadao Asami, Masaru Tanokura

**Affiliations:** 1 Department of Applied Biological Chemistry, Graduate School of Agricultural and Life Sciences, The University of Tokyo, 1-1-1 Yayoi, Bunkyo-ku, Tokyo 113-8657, Japan; 2 JST, CREST, 4-1-8 Honcho, Kawaguchi, Saitama 332-0012, Japan; 3 Department of Biochemistry, King Abdulaziz University, Jeddah 21589, Saudi Arabia

## Abstract

The perception of two plant germination inducers, karrikins and strigolactones, are mediated by the proteins KAI2 and D14. Recently, KAI2-type proteins from parasitic weeds, which are possibly related to seed germination induced by strigolactone, have been classified into three clades characterized by different responses to karrikin/strigolactone. Here we characterized a karrikin-binding protein in *Striga* (ShKAI2iB) that belongs to intermediate-evolving KAI2 and provided the structural bases for its karrikin-binding specificity. Binding assays showed that ShKAI2iB bound karrikins but not strigolactone, differing from other KAI2 and D14. The crystal structures of ShKAI2iB and ShKAI2iB-karrikin complex revealed obvious structural differences in a helix located at the entry of its ligand-binding cavity. This results in a smaller closed pocket, which is also the major cause of ShKAI2iB’s specificity of binding karrikin. Our structural study also revealed that a few non-conserved amino acids led to the distinct ligand-binding profile of ShKAI2iB, suggesting that the evolution of KAI2 resulted in its diverse functions.

Strigolactones (SLs) are originally isolated as germination stimulants for parasitic weeds *Striga* and *Orobanche* genera^1^, which are among the most severe threats for agricultural production in sub-Saharan Africa^2^. Later, it has been proved that SLs also act as symbiotic signals by triggering hyphal branching of arbuscular mycorrhizal fungi^3^, and as endogenous phytohormone by inhibiting lateral branching^4^,^5^. In addition to SLs, karrikins^6^,^7^, which are abiotic butenolide derived from burning vegetation, can also induce seed germination after forest fires^8^. Interestingly, these two distinct classes of germination stimulus, SLs and karrikins, adopt similar structures sharing a common lactone ring, which is supposed to be vital for signal perception^9^.

SL receptor D14 (DWARF14) and karrikin-responding protein KAI2 (KARRIKIN INSENSITIVE 2) are closely related homologues belonging to α/β hydrolases superfamily. Another convergent point of these two classes of proteins is that they both need the F-box protein, MAX2 (MORE AXILLARY GROWTH2)^10^ or D3 (DWARF3) in *Oryza sativa*^11^, for signal transduction through direct^12^,^13^,^14^ or indirect interaction^15^. Despite the fact that D14 and KAI2 share a lot in common, they have different functions and play distinct roles in regulation of plant growth. D14 proteins are verified to be capable of binding and hydrolyzing GR24 (synthetic SL analogue) with conserved catalytic triad residues (Ser-His-Asp). In contrast, KAI2 protein is able to bind both GR24 and karrikin and shows hydrolytic activity toward GR24^16^, while no detectable hydrolytic activity toward karrikin has been reported so far. Besides, D14 is mainly involved in inhibiting axillary bud outgrowth through perception of SLs while KAI2 is required for seed germination by perception of karrikins and/or exogenous SLs and for early seedling development by mediating responses to SL^17^.

This raised questions about how these two highly similar proteins show different patterns of ligand perception. Recently, KAI2 paralogs from parasitic weeds have been phylogenetically classified into three clades (ancestral, intermediate- and fastest-evolving KAI2) with different responses to karrikin/SL^18^,^19^ (Supplementary Figure S1). Ancestral/conserved clade is most conserved to KAI2 phylogenetically but nonresponsive to neither karrikin nor SL; diverse/fastest-evolving clade is corresponding to D14 being responsive to SL but not karrikin; while intermediate clade is responsive to karrikin but not SL. Since crystal structures of KAI2 and D14 have already been reported^12^,^20^,^21^,^22^,^23^,^24^,^25^, the structural study of KAI2 of intermediate clade would deepen our understanding of the molecular mechanism underlying these ligand-binding specificities. Here we present the crystal structure of *S. hermonthica* intermediate KAI2 (ShKAI2iB), which has 98% sequence identity with ShKAI2i (Supplementary Figure S2) that has been reported to respond to karrikin but not to GR24 through complementation experiments^18^.

## Results

### Ligand-binding specificity of ShKAI2iB

**Figure 1:**
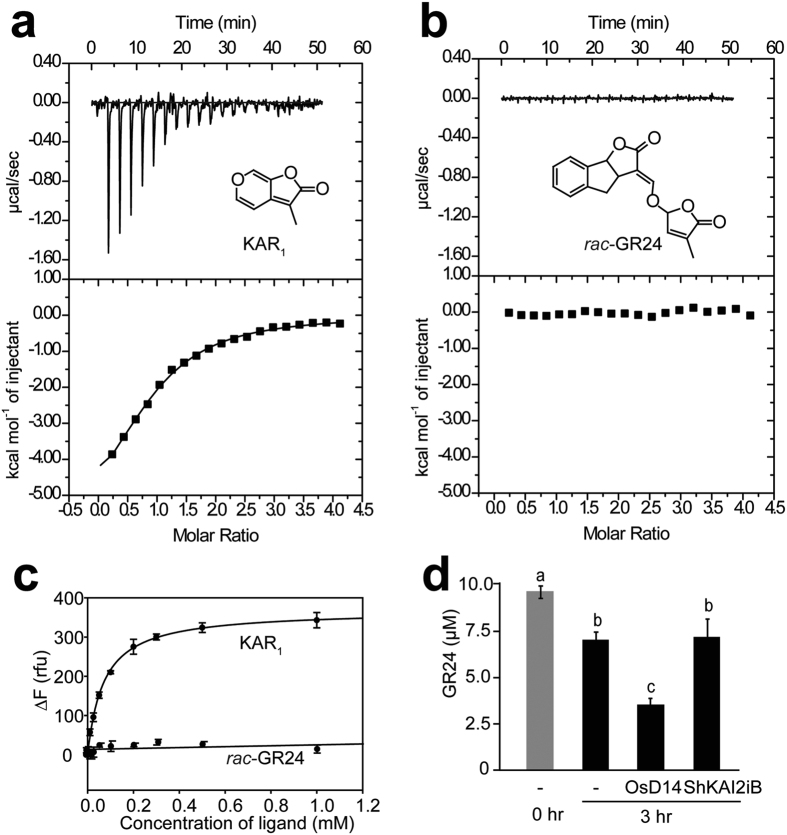
Binding specificity of ShKAI2iB. (**a**,**b**) Results of ITC experiments of ShKAI2iB titrated with KAR_1_ (**a**) and *rac*-GR24 (**b**). Binding of KAR_1_ to ShKAI2iB exhibited a *K*_D_ value of 77.6 ± 3.4 μM, along with Δ*H* (enthalpy change) of −6.48 ± 0.4 kcal mol^–1^, Δ*S* (entropy change) of –3.99 cal mol^–1^ deg^–1^ and N (number of sites) of 0.91 ± 0.05 (Supplementary Figure S4 and Table S2). (**c**) Changes of fluorescence intensity by the addition of KAR_1_ and *rac*-GR24 to ShKAI2iB. Intrinsic fluorescence was recorded at excitation wavelength of 285 nm and emission wavelength of 333 nm. Fitting using SigmaPlot 13.0 indicated that *K*_D_ value was 70.0 ± 3.4 μM (*n* = 3) for KAR_1_ binding to ShKAI2iB. (**d**) Enzymatic degradation assay of *rac*-GR24. The data means ± SE of three independent experiments. Statistical differences between the groups were calculated with ANOVA analysis followed by Tukey–Kramer test. Bars with different letters are significantly different with p < 0.01.

ITC (isothermal titration calorimetry) experiments along with intrinsic fluorescence assays were used to determine the binding properties of ShKAI2iB towards KAR_1_ (3-methyl-2*H*-furo [2,3-*c*] pyran-2-one, a member of the karrikin family) and *rac*-GR24 (synthetic SL analogue). The results of the ITC experiments involving ShKAI2iB and KAR_1_ showed a dissociation constant (*K*_D_) of 77.6 ± 3.4 μM, and the intrinsic fluorescence assays showed a *K*_D_ of 70.0 ± 3.4 μM (Fig. 1a–c). However, no detectable heat change or fluorescence change were observed after adding *rac*-GR24 to ShKAI2iB. In addition, our hydrolysis assay with HPLC (Fig. 1d) indicated that ShKAI2iB was not capable of hydrolyzing *rac*-GR24, although it has been reported that KAI2 is able to bind both KAR_1_ and GR24 and exhibits hydrolytic activity toward GR24^16^. Our results indicated a novel binding specificity in which ShKAI2iB bound karrikin but not SL, in agreement with the reported results of cross-species complementation assays of *ShKAI2i*^18^.

### Overall structure of ShKAI2iB

**Figure 2:**
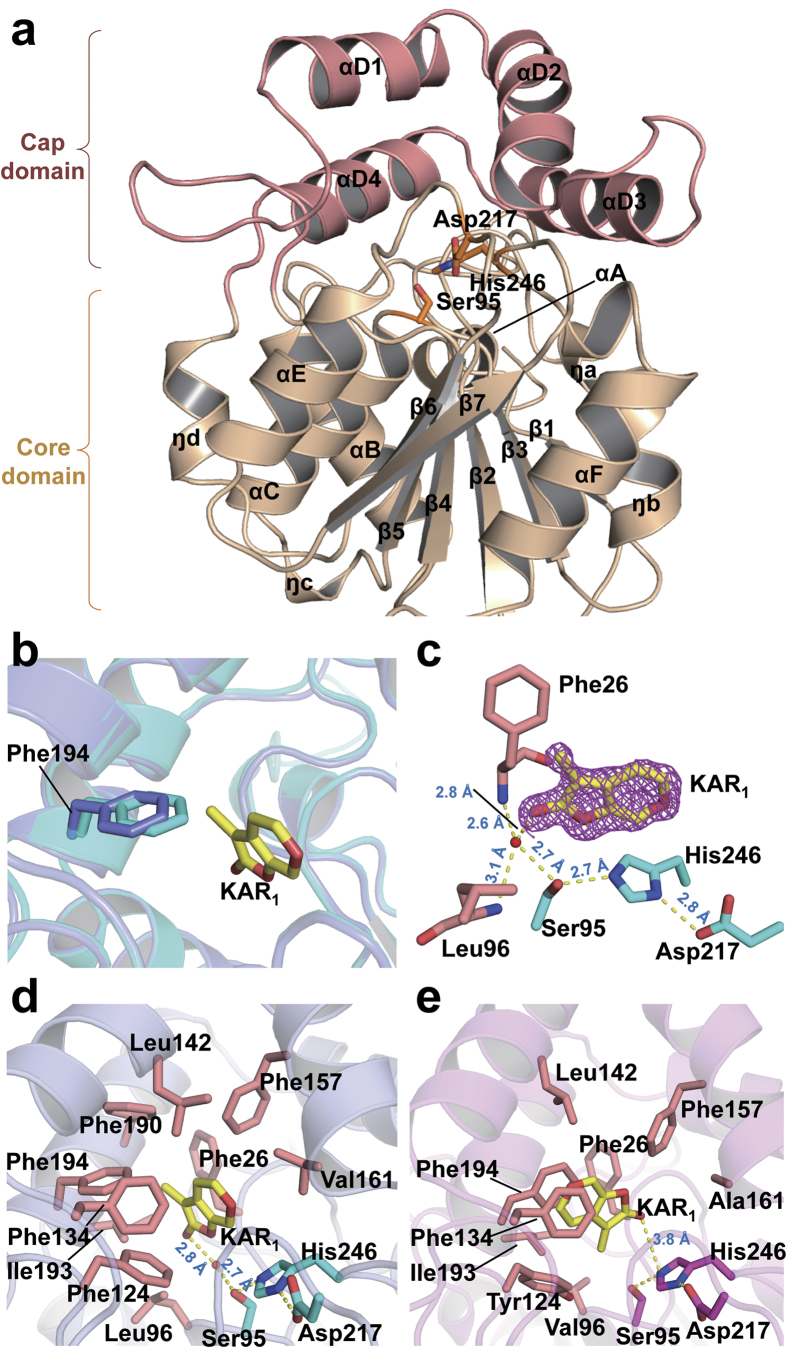
Structures of ShKAI2iB and its KAR_1_ complex. (**a**) Structure overview of ShKAI2iB in a ligand-free (apo) state. Cap domain and core domain are colored salmon and wheat, respectively. Catalytic triad residues are indicated as cyan sticks. (**b**) Structural alignment of apo- (cyan) and KAR_1_-bound ShKAI2iB (purple). Phe194 and KAR_1_ (yellow) are highlighted in stick model. (**c**) Hydrogen-bonding network between KAR_1_ and the catalytic residues of ShKAI2iB. KAR_1_ is shown by a yellow stick and contoured 2*F*_o_-*F*_c_ map (Pink mesh) at level of 1.0σ. Catalytic triad residues are highlighted in cyan sticks and the residues for oxyanion hole are in salmon sticks. Red sphere represents water molecule. Hydrogen bonds and their lengths are represented using dashed lines and blue values (Å), respectively. (**d**,**e**) Binding modes of KAR_1_ in the cavity of ShKAI2iB (**d**) and KAI2 ((**e)** PDB ID 4JYM). Residues surrounding KAR_1_ (yellow) are highlighted in salmon sticks. Catalytic triad residues of ShKAI2iB are shown as cyan sticks and those of KAI2 are shown in magenta sticks. Red spheres stand for water molecules. Hydrogen bonds are represented by yellow dashes and lengths are shown with blue values (Å).

To determine why ShKAI2iB exhibited a different binding specificity from KAI2 and D14s, we solved crystal structures of apo ShKAI2iB at 2.0-Å resolution and ShKAI2iB-KAR_1_ complex at 1.2-Å resolution (Supplementary Figure S3 and Table S1). ShKAI2iB consisted of a core domain and a cap domain (Fig. 2a). The core domain, also known as α/β hydrolase domain^26^, was composed of seven strands (β1–β7), five helices (αA, αB, αC, αE and αF) and four 3_10_ helices (ŋa, ŋb, ŋc and ŋd) as shown in Fig. 2a. The cap domain was composed of 4 tandem helices, which forms two antiparallel V shapes (αD1-αD2 and αD3-αD4) stabilized mainly by hydrophobic interactions between overlapping helices. Meanwhile, this cap domain was connected to the core domain by loops β5-αD1 and αD4-ŋd, and contacted by loops β2-ŋa, β3-ŋc and β7-αF through hydrogen bonds and hydrophobic interactions. A cavity was formed between the two domains, in common with other KAI2/D14 proteins reported^12^,^20^,^21^,^22^,^23^,^24^,^25^, and catalytic triad (Ser95-His246-Asp217) lied at the bottom of the cavity.

### KAR_1_-binding mode of ShKAI2iB

Comparing crystal structures of apo and KAR_1_-bound ShKAI2iB, no significant structural changes were observed, except Phe194 in the cavity (Fig. 2b). Upon KAR_1_ binding, Phe194 moved 1 Å away from KAR_1_ binding site, creating a space to accommodate KAR_1_. Interestingly, Phe194 was also the only residue in the cavity that possessed conformational change for D-OH-bound OsD14^23^ and KAR_1_-bound KAI2^21^. However, F194A mutation showed no significant change in the results of our ITC assays (Supplementary Figure S4 and Table S2), indicating that its phenyl group was not required to capture KAR_1_ although it could be moved to expand the KAR_1_-binding site.

In the complex structure of ShKAI2iB-KAR_1_, KAR_1_ was embedded completely in the cavity, with the oxygen-bearing edge facing down, and thus, the carbonyl group of KAR_1_ pointing toward the bottom of the cavity (Fig. 2c). Carbonyl oxygen of KAR_1_ in the ShKAI2iB-KAR_1_ structure was able to form hydrogen bond with the hydroxyl group of Ser95 through a water molecule. This water molecule also formed hydrogen bonds with main-chain amide nitrogen atoms of Phe26 and Leu96, which acts as an oxyanion hole in catalytic activity of α/β hydrolase. Moreover, methyl group of KAR_1_ (ShKAI2iB) was embedded in the hydrophobic side consisting of Phe26, Phe190, Ile193 and Phe194. Actually, S95A exhibited almost no heat change following the addition of KAR_1_ in the ITC experiment (Supplementary Figure S4), indicating that Ser95 is vital for KAR_1_ binding, likely by stabilizing a water molecule in the vicinity. In consistent with this observation, Ser95 has been reported to be essential for normal seedling karrikin responses of *Arabidopsis* KAI2^27^.

On the other hand, the methyl group of KAR_1_ was embedded in the hydrophobic side consisting of Phe26, Leu142, Phe190, Ile193 and Phe194, and the pyran ring of KAR_1_ formed face-to-edge aromatic-dipole interactions and hydrophobic interactions with ShKAI2iB. The results of our ITC assays showed that L142A and F190L mutants exhibited a two-fold decrease in affinity for KAR_1_ with *K*_D_ value of 196 ± 24 μM and 186 ± 43 μM, respectively (Supplementary Figure S4 and Table S2). In addition, a Val139 mutation in Leu, a residue that interacts with KAR_1_ in KAI2, showed two-fold higher affinity (*K*_D_ value of 36 ± 7 μM), suggesting that Val139 might strengthen the hydrophobic interaction with KAR_1_. Consequently, KAR_1_ fit in the cavity of ShKAI2iB and was stabilized by both hydrogen bonds and hydrophobic interactions.

### Structural bases for KAR_1_ binding

**Figure 3:**
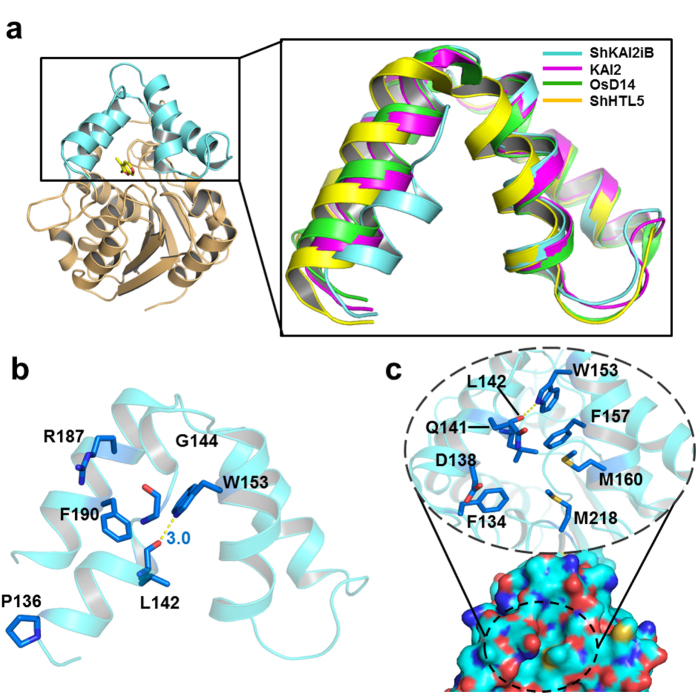
Structural basis for regulating the conformation of cap domain. (**a**) Superposition of cap domain from ShKAI2iB in a ligand-free (apo) state (cyan) and KAI2 (apo, PDB ID 4JYP) (magenta), OsD14 (PDB ID 3WIO) (Green) and ShHTL5 (PDB ID 5CBK) (Yellow). (**b**) Regulatory residues in the cap domain of ShKAI2iB (ligand-free apo state). Blue sticks represent the residues involved in closed conformation of helix αD1. Hydrogen bond between Leu142 and Trp153 was indicated by yellow dashed line and bond length was shown in blue value (Å). (**c**) Surface representation (bottom) and residues around cavity entry of ShKAI2iB (top). Cyan, blue, red, and yellow surfaces represent carbon, nitrogen, oxygen, and sulfur atoms, respectively. In the dashed circle, some residues of helix αD1 and αD2, which are supposed to be the access to KAR_1_ binding pocket, are emphasized in close-up view. The entrance is closed and inaccessible to solvent.

However, the KAR_1_-binding mode of ShKAI2iB was different from that of KAI2. In the KAI2-KAR_1_ structure (PDB ID 4JYM), KAR_1_ was upside down with the methyl group pointing toward the catalytic triad instead and was located at a slightly distal position from Ser95 (Fig. 2e)^21^. To investigate the structural basis for different binding mode of KAR_1_ in ShKAI2iB and KAI2, we compared the structure of ShKAI2iB with KAI2. Root-mean-square deviation (RMSD) between the two structures was 0.8 Å for 262 C_α_ atoms superimposed. In spite of the overall structural similarity as well as high sequence identity as mentioned above, there were some structural differences between ShKAI2iB and KAI2. Superposition of ShKAI2iB with other KAI2 and D14 showed that inward shift occurred on helix αD1 of ShKAI2iB, which subsequently narrowed the KAR_1_-binding pocket of ShKAI2iB (Fig. 3a). There are four major structural bases for this inward shift of helix αD1. In the structure of ShKAI2iB, Pro136 was located at the N-terminus of helix αD1, as opposed to Gln136 in the structure of KAI2, resulting in a sharper turn than that of KAI2, which forces helix αD1 to lean towards helix αD2 (Fig. 3b). Meanwhile, helix αD1 has the highly flexible amino acid Gly144 at its C-terminus, which disrupts helix αD1. In addition, two bulky residues in helix αD4, Arg187 and Phe190, extrude helix αD1 toward the cavity. Consequently, the helix αD1 approached to helix αD2, and a hydrogen bond forms between the side-chain amine of Trp153 and the main-chain carbonyl group of Leu142, which was exposed due to the disruption of helix formation by Gly144.

As a result of inward shift of helix αD1, the side chain of Leu142 will cause a steric clash with KAR_1_ if it takes the same orientation as that in the KAI2-KAR_1_ structure. Besides, the side-chain rotation of Phe194 is required to sandwich KAR_1_ between Phe194 and Phe134 in KAI2, whereas the same rotation cannot occur in ShKAI2iB because the rotated side-chain will sterically clash with the bulky Phe190 (Fig. 2d). Phe190 is a characteristic residue of ShKAI2iB that is substituted by a Gly residue in KAI2 (Gly190), and appears to take part in reinforcing the hydrophobic interaction of Leu142 and Phe194 with KAR_1_. These structural bases form a small cavity that precisely enough to encapsulate KAR_1_. As a result, compared with the cavity sizes of KAI2 (238 Å^3^), OsD14 (461 Å^3^) and ShHTL5 (713 Å^3^), the cavity of ShKAI2iB is the smallest: 155 Å^3^. This also explains why ShKAI2iB cannot accommodate SL, which is larger than karrikin.

### Conformational changes of cap domain

**Figure 4:**
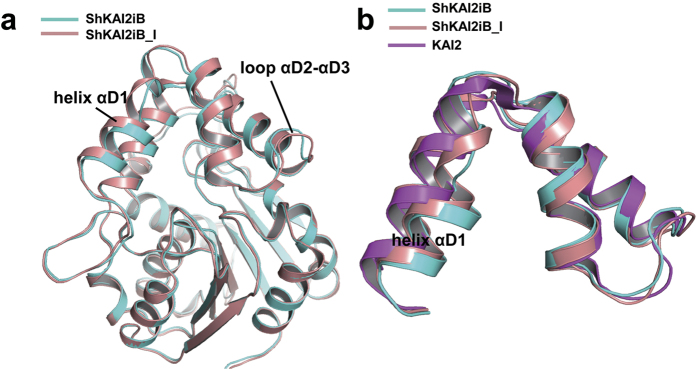
Structural alignment of two different conformations of ShKAI2iB. (**a**) Superposition of two different conformations of ShKAI2iB in the ligand free (apo) state, ShKAI2iB (cyan) and ShKAI2iB-I (intermediate, salmon). Conformational changes between ShKAI2iB and ShKAI2iB-I are observed on helix αD1 and loop αD2-αD3 of cap domain. (**b**) Cap domain of ShKAI2iB in two states and KAI2 (apo, PDB ID 4JYP).

Another outcome derived from the inward shift of helix αD1 is the closing of the entrance to the ligand-binding cavity of ShKAI2iB (Fig. 3c). This shift of helix αD1 has been reinforced by hydrogen bond between Leu142 and Trp153. Moreover, the residues with bulky side chains, such as Phe134, Asp138, Gln141, Leu142, Phe157, Met160 and Met218, are located around the entrance and therefore clog the hydrophobic cavity. Unexpectedly, the complexed structure of ShKAI2iB with KAR_1_ also has a closed cavity, indicating that conformational changes of the cap domain are required to enable KAR_1_ to access the cavity. We suggest that helix αD1 might be the gate-keeper because different conformation of helix αD1 was observed in another ligand-free structure of ShKAI2iB [ShKAI2iB-I (intermediate)] during our attempt to acquire complex of ShKAI2iB with KAR_1_. Superposed structures of ShKAI2iB and ShKAI2iB-I showed that helix αD1 moved outwardly and caused conformational changes on the residues Leu142–Gly144 (Fig. 4). To compare the flexibility of helix αD1, we calculated the normalized *B*-factors by dividing every *B*-factors of helix αD1 by the average *B*-factors of overall structures. The normalized *B*-factors of helix αD1 are 1.3 for all three structures, suggesting that no significant difference in flexibility of helix αD1 between the three structures and helix αD1 is slightly more flexible than other part. Although the cavity of ShKAI2iB-I was also closed, the entrance was slightly open. These structures in the ligand-free state imply that helix αD1 adopts multiple conformations in solution. The addition of KAR_1_ might shift the equilibrium towards the ligand-binding conformation with a wider entry to the hydrophobic cavity. Therefore, we suggest that helix αD1 of ShKAI2iB might be allosterically involved in ligand binding and ShKAI2iB closed the gate again and locked KAR_1_ after capturing KAR_1_.

## Discussion

A recent report indicates that *KAI2* paralogues from *S. hermonthica* can be divided into three types: *KAI2c* clade that is most conserved with *AtKAI2*; *KAI2d* clade that is most diversely evolved and most likely to be involved in SL perception in *S. hermonthica* and *KAI2i* clade that is intermediate between *KAI2c* and *KAI2d*^18^. Among them, *ShKAI2i* is a *KAI2* paralogue that responses to only karrikin for *Arabidopsis* seed germination through complementary assays. In the present study, we identified the intermediate KAI2-like protein with distinct characteristic from KAI2 and D14. According to our results and previous results of cross-species complementation assays of *KAI2*^18^, ShKAI2iB was unable to bind and hydrolyze GR24 but capable of binding karrikin. Our structural study revealed that a few non-conserved amino acids led to the distinct ligand-binding profile of ShKAI2iB, supporting that evolution of KAI2 resulted in diverse functions of KAI2.

ShKAI2iB exhibited a different KAR_1_ binding mode from KAI2, by forming hydrogen bond between Ser95 and KAR_1_ through a water molecule, which means that Ser95 contributes to capturing karrikin but not hydrolysis of KAR_1_, because a covalent bond between Ser and carbonyl carbon of substrate is required for catalytic reaction. In fact, hydrolytic activity of KAI2 for KAR_1_ has not been reported. On the other hand, catalytic triad residues of D14/KAI2 proteins are not only important for enzymatic activity, but also necessary for interaction with other proteins or degradation-mediated feedback regulation^12^,^23^,^28^,^29^. For example, catalytic Ser residue is necessary for the interaction between DAD2 and PhMAX2A, a MAX2 orthologue from *P. hybrida*^12^. In the case of KAI2, both karrikin-induced and GR24-induced degradation of KAI2 has been observed in a Ser95-dependent manner^29^; however, it remains unclear whether catalytic reaction occurs in the karrikin perception of KAI2. Our results suggest that the catalytic Ser residue (Ser95) of ShKAI2iB functions to capture KAR_1_ at the active site, which might contribute to activating downstream signaling without catalytic reaction. In the crystal, the helix αD1 is near (about 3.5 Å) a helix (helix αF) from neighbor molecule; therefore, it is not excluded that the crystal contact affects the position of the helix αD1. However, ShKAI2iB should not take the same position of helix αD1 and bind KAR_1_ in the same mode as AtKAI2. If so, KAR_1_ is distal from Ser95, which conflicts with our ITC data that substitution of Ser95 to Ala abolishes binding. Although there are two other interactions from the main chain, Ser95 may be crucial for the overall network of KAR_1_ binding by stabilizing both the water molecule and the assumed catalytic residues His246 and Asp217.

In addition to the different recognition mode of catalytic triad Ser95, KAR_1_ exhibited other different binding characteristics in the catalytic cavity of ShKAI2iB and KAI2. In the latter case, KAR_1_ located at the outer entrance of KAI2’s cavity and possibly served as new interface for partner recognition^21^. In the structure of KAR_1_-bound ShKAI2iB, the cavity was closed and KAR_1_ was unable to be exposed to the solvent. Therefore, a distinct signal might be transferred by ShKAI2iB. Nonetheless, it was reported that *ShKAI2i* could be complementary to *Arabidopsis KAI2* for germination induction by KAR_1_. Therefore, the recognition of KAR_1_ might be loose for signal transfer. On the other hand, this difference might imply a distinct function/response of ShKAI2iB in *S. hermonthica*. In fact, KAR_1_ failed to induce germination of *S. hermonthica*^18^,^30^, suggesting that KAR_1_ binding of ShKAI2iB might not be a signal of germination. Instead, it could be a sign that no hosts are near, which would keep the seeds dominant considering that karrikin is derived from smoke after forest fires. Interestingly, the transcripts of ShKAI2iB decreased during conditioning of *Striga* seeds (Supplementary Figure S5). This expression profile might suggest that ShKAI2iB functions to suppress of seed germination. There might also be other authentic endogenous ligands for ShKAI2iB as suggested for KAI2 previously^27^. Our structural evidences provide the ligand-binding specificity of ShKAI2iB toward KAR_1_, guiding the exploration of authentic endogenous ligands. On the other hand, according to the phylogenetic analysis (Supplementary Figure S1), there are other KAI2/D14 orthologues in *S. hermonthica*, some of which might be authentic SL receptor. Our study revealed that the helix αD1 was a key factor for ligand-binding specificity, providing additional information for discriminating SL receptor in *S. hermonthica*.

## Methods

### Overexpression and purification of recombinant proteins

The coding sequence cDNA of ShKAI2iB was amplified by PCR using total complementary DNA from the total RNA of 1-day conditioned *Striga* seeds. ShKAI2iB (1–270, C270S) was designed for crystallization and other assays. For expression in *Escherichia coli*, the PCR product was cloned into an expression vector pGEX-6P-3 (GE Healthcare) and subsequently transformed into *E. coli* strain Rosetta (DE3) (Novagen). IPTG-induced overexpression was performed for 20 h at 25 °C. For purification, cell pellets were lysed by sonication in buffer A (20 mM Tris-HCl, pH 8.0, 0.3 M NaCl, 1 mM DTT), and the soluble fraction separated by centrifugation was purified using Glutathione Sepharose 4B resin (GE Healthcare) and a Resource Q anion-exchange column (GE Healthcare). ShKAI2iB mutants were overexpressed and purified with the same procedure as wild-type protein. For crystallization, buffer exchange accompanied by concentration was performed using Vivaspin 20 (5,000 MWCO PES) (Sartorius). Purified ShKAI2iB was dialyzed against buffer B (20 mM HEPES-NaOH, pH 8.0, 50 mM NaCl) for the ITC experiments and the intrinsic fluorescence assay.

### Isothermal titration calorimetry

Binding assays of ShKAI2iB and KAR_1_ were performed using a MicroCal iTC_200_ isothermal titration calorimeter (GE Healthcare). Prior to the ITC experiments, concentrated ShKAI2iB was dialyzed against a buffer consisting of 20 mM HEPES-NaOH, pH 8.0, and 50 mM NaCl to remove dithiothreitol and then adjusted to a final concentration of 150 μM. The sample cell was filled with ShKAI2iB solution (204 μl). Two microliters of 3 mM KAR_1_ or 3 mM *rac*-GR24 (equimolar mixture of two enantiomers: GR24^5DS^ and GR24^*ent*-5DS^) was injected into the prepared protein solution by 20 consecutive 2.0 μl aliquots at 150 s intervals at 10 °C. The first injection volume was 0.4 μl, and the observed thermal peak was excluded from the data analyses. Duplicate experiments were performed independently. A negative control was made by titrating 3 mM KAR_1_ into a buffer (20 mM HEPES, pH 8.0, 50 mM NaCl) in the same manner. Data fitting was performed using Origin software in the “one set of sites” mode. The dissociation constant (*K*_D_) values were calculated from duplicate thermograms (mean ± S.D.).

### Intrinsic fluorescence assay

*K*_D_ values were also determined by using intrinsic fluorescence assays. Fluorescence measurements were conducted as previously described^16^ with minor modifications. One microliter of KAR_1_ or *rac*-GR24 dissolved in DMSO was added to 100 μl of 10 μM protein solution to reach a certain concentration. Flat-bottomed, black 96 well plates were used to read fluorescence intensity using a Tecan Infinite M1000 monochromator. Measurements were taken at room temperature under a 285 nm excitation wavelength, a 333 nm emission wavelength, 50 flashes, and a 400 Hz flash frequency with a gain of 70 and a 2 μs integration time. Δ*F* (rfu, relative fluorescence units) was calculated by subtracting the fluorescence of the DMSO control. SigmaPlot 13.0 was used to fit and determine *K*_D_ values with a one-site saturation model.

### Enzymatic degradation assay

The enzymatic degradation assay of *rac*-GR24 was performed in a total volume of 1 ml of PBS buffer containing 10 μM *rac*-GR24. Purified OsD14^23^ and ShKAI2iB were added at a final concentration of 6 μg ml^−1^ and incubated for 3 h at 37 °C. Then 100 mg of NaCl and 100 μl of 0.1 M HCl were added to each reaction solution, and the reaction solutions were extracted with 400 μl of ethyl acetate three times. The ethyl acetate layers were combined and dried in vacuo and dissolved in 50 μl of methanol. For each layer, 10 μl was applied to the HPLC analyses. The reverse-phase chromatographic separation was performed on a Jasco HPLC system that was equipped with an HPLC pump of model PU 2080 (Jasco) and a photodiode array detector MD1510 (Jasco). The system was controlled by the ChromNAV (Ver. 1.18.07) software program (Jasco). The analytical column was a CAPCELL CORE C18 (Φ 4.6 × 100 mm, Shiseido). The analytes were eluted under gradient conditions using methanol ramped linearly to 90% methanol at 9 min and held for 4.5 min before resetting to the original conditions. The contents of *rac*-GR24 was calculated by the peak area at the retention time 6.2 min with the regression equation obtained from the calibration curve produced using a dilution series of *rac*-GR24 solution. Statistical analysis was performed by using the JMP11 software (SAS Institute Inc.). Statistical differences between the groups were calculated with ANOVA analysis followed by Tukey–Kramer test.

### Crystallization, data collection, structure determination and refinement

Crystals were obtained using 7.4 mg ml^−1^ of ShKAI2iB protein with reservoir solution consisting of 100 mM Tris (pH 7.5) and 3 M sodium formate with sitting-drop vapor diffusion method at 4 °C. 3–4 weeks were necessary for the crystals to grow. Crystal of ShKAI2iB was picked up and soaked with the reservoir solution containing 25% (v/v) ethylene glycol as cryoprotectant before mounting. A diffraction data set was collected in a nitrogen cryostream of 95 K using an in-house Rigaku R-AXIS VII imaging-plate detector (Rigaku, Japan). The diffraction data were indexed, integrated and scaled using the XDS program^31^. The crystal belonged to space group *P*6_1_22 possessing unit-cell parameters *a* = *b* = 75.9, *c* = 181.5 Å. Mathews coefficient was estimated to be 2.53 Å^3^ Da^−1^ and solvent content was 51.3%^32^, suggesting that there was one molecule in asymmetric unit. Molecular replacement was carried out using MOLREP^33^ of CCP4 program suite and the crystal structure of OsD14 (PDB ID 3VXK) as a template. BUCCANEER^34^ was applied for automatic model building. Refinement was performed using REFMAC5^35^ and WINCOOT^36^ to a final *R*_work_ of 18.0% and *R*_free_ of 22.5%. PyMOL viewer (Version 1.5.0.4 Schrödinger, LLC) was used to depict all the structures and CASTp server^37^ was used to calculate volume of protein cavities using probe radius of 2.0 Å. During attempt of crystallization of ShKAI2iB with 2.5 mM KAR_1_, crystal structure of ShKAI2iB-I was solved using structure of ShKAI2iB as template model and final model was refined to *R*_work_ of 20.7% and *R*_free_ of 26.6%. No electron density of KAR_1_ has been observed. Complexed structure of ShKAI2iB with KAR_1_ was solved with soaking in addition to co-crystallization. Since the binding affinity of KAR_1_ to ShKAI2iB was slightly weak, we tried the crystallization of ShKAI2iB with the KAR_1_ concentration of 10 mM. Acquired crystal was soaked with 30 mM KAR_1_ for 5 hours and used for diffraction data collection. The collected data were indexed, integrated and scaled with *HKL*-2000^38^. Crystal structure of KAR_1_-bound ShKAI2iB was solved by molecular replacement with the model of ShKAI2iB and finally refined to resolution of 1.2 Å with *R*_work_ of 14.1% and *R*_free_ of 15.5%. *B*-factors for each structure were calculated with Baverage^39^, and the root-mean-square deviation (RMSD) between the two structures was calculated using a Dali server^40^. Data collection and refinement statistics are summarized in Supplementary Table S1.

### Sequence alignment and phylogenetic tree

CLUSTAL W^41^ was used for multiple sequence alignment with default parameters, and the result was displayed by ESPript 3.0^42^. Aligned sequences included ShKAI2iB, KAI2 (*A. thaliana* KAI2, NCBI GI: 15235567), OsD14 (*O. sativa* D14, NCBI GI: 115451411), AtD14 (*A. thaliana* D14, NCBI GI: 75337534) and DAD2 (*P. hybrida* D14, NCBI GI: 404434487). Blastp searches were performed using the amino acid sequence of ShKAI2iB as a query against *O. sativa* and *A. thaliana* genus using non-redundant GenBank databases. Results were filtered using cut-off *E* value of 1e^−8^. EST sequences and genome sequences of *S. hermonthica* were investigated in the *S. hermonthica* EST Database (http://striga.psc.riken.jp/est2uni/) and Parasitic Plant Genome Project (http://ppgp.huck.psu.edu/). Sequences were further filtered and screened for truncation and duplication. Phylogenetic tree was created using MEGA version 6^43^ with UPGMA method.

### *Striga* germination

Seeds of *S. hermontica* harvested in Sudan were kindly provided by Prof. A.E. Babiker (Sudan University of Science and Technology) and imported with the permission from the Minister of Agriculture, Forestry and Fisheries. The *Striga* germination assay was performed as described previously^44^. *Striga hermontica* seeds were surface sterilized and pre-incubated (conditioned) on glass paper disks placed on distilled water-satured filter paper at 30 °C. Then seeds were treated with 0.1 μM of GR24. After further incubation at 30 °C for 3 days, GR24-treated seeds were microscopically evaluated for germination.

### Relative expression levels of the *ShKAI2iB* gene

Total RNA was extracted from conditioned seeds before GR24-treatment, purified with the Total RNA Extraction Mini Kit (RBC Bioscience), and converted to cDNA with the PrimeScript RT Reagent Kit (Takara Bio) according to the manufacturer’s protocols. Quantitative PCR was performed with SYBR Premix Ex Taq (Takara Bio) and the Thermal Cycler Dice Real Time System TP800 (Takara Bio). The transcript levels of *ShKAI2iB* were normalized against those of *ShUBQ1*^45^, using primers specific for *ShKAI2iB* (5′-TAGGGTCGGTGGAAGGTCAGTC-3′ and 5′-CAGCACTGGGATGGCAACCT-3′), and *ShUBQ1* (5′-CATCCAGAAAGAGTCGACTTTG-3′ and 5′-CATAACATTTGCGGCAAATCA-3′). Student’s *t*-test was used to determine the significance of differences relative to the transcript level in *Striga* seeds conditioned for 1 day.

## Additional Information

**Accession code**: The atomic coordinates and structure factors have been deposited in the Protein Data Bank (PDB) under accession code 5DNW, 5DNV and 5DNU for ShKAI2iB, ShKAI2iB-I and KAR1-bound ShKAI2iB, respectively.

**How to cite this article**: Xu, Y. *et al*. Structural basis of unique ligand specificity of KAI2-like protein from parasitic weed *Striga hermonthica*. *Sci. Rep*. **6**, 31386; doi: 10.1038/srep31386 (2016).

## Supplementary Material

Supplementary Information
