# Supplementary material for: Structural basis of unique ligand specificity of KAI2-like protein from parasitic weed Striga hermonthica
Source: Sci Rep. 2016 Aug 10;6:31386. doi: 10.1038/srep31386 (PMC4979206; doi:10.1038/srep31386)
Supplement: Supplementary Information [file srep31386-s1.pdf]

Supplementary Information for

Structural basis of unique ligand specificity of KAI2-like protein from parasitic weed *Striga hermonthica*

Yuqun Xu[1], Takuya Miyakawa[1], Hidemitsu Nakamura[1], Akira Nakamura[1], Yusaku Imamura[1], Tadao Asami[1,2,3], Masaru Tanokura[1,*]

Affiliations:

[1] Department of Applied Biological Chemistry, Graduate School of Agricultural and Life Sciences, The University of Tokyo, 1-1-1 Yayoi, Bunkyo-ku, Tokyo 113-8657, Japan

[2] JST, CREST, 4-1-8 Honcho, Kawaguchi, Saitama 332-0012, Japan

[3] Department of Biochemistry, King Abdulaziz University, Jeddah 21589, Saudi Arabia

*Corresponding author. Email: amtanok@mail.ecc.u-tokyo.ac.jp

Contents

Supplementary Figure S1: Phylogenetic tree of KAI2 and D14 family proteins.

Supplementary Figure S2: Multiple sequence alignment of KAI2/D14 proteins.

Supplementary Figure S3: Electron density of ShKAI2iB and its complex with KAR_1.

Supplementary Figure S4: Results of ITC experiments between ShKAI2iB mutants and KAR_1.

Supplementary Figure S5: Transcript level of *ShKAI2iB* during *Striga* seed conditioning.

Supplementary Table 1: Statistics of X-ray collection data and refinement.

Supplementary Table 2: Thermodynamic parameters determined by ITC.

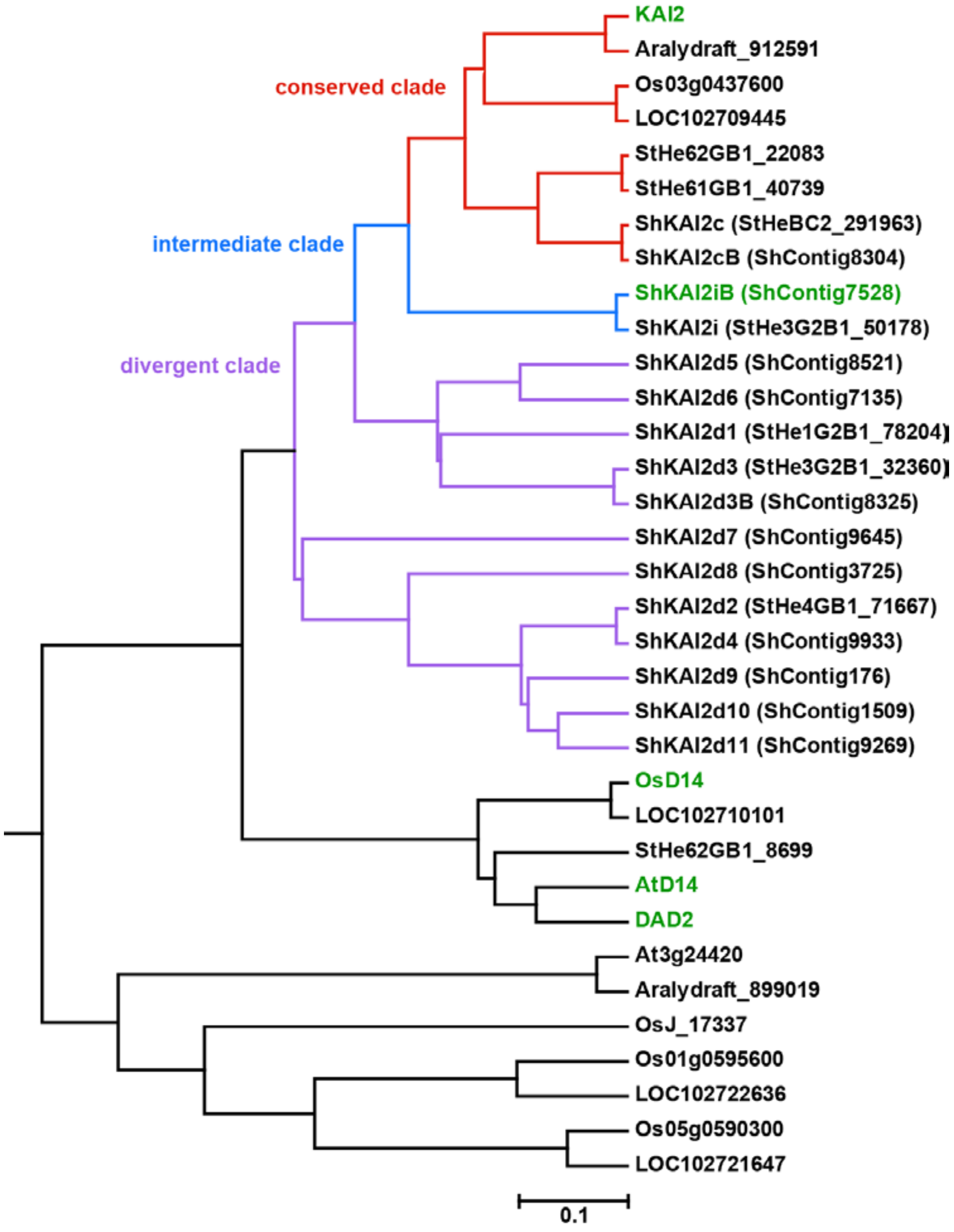

Supplementary Figure 1 Phylogenetic tree of KAI2 and D14 family proteins. UPGMA phylogenetic tree was generated with amino acid sequences of D14 and D14L proteins among *Oryza* and *Arabidopsis*, *S. hermonthica* and that of *Petunia hybrida* DAD2. *Arabidopsis* KAI2 protein is able to bind both karrikin and SL and D14 is able to bind SL specifically. Branch length represents substitutions per site. Genes with accession numbers started with ShContig were quoted from *S. hermonthica* EST Database (http://striga.psc.riken.jp/est2uni/) and those started with StHe were from Parasitic Plant Genome Project (http://ppgp.huck.psu.edu/).

Different clades of KAI2 paralogues were divided according to a previous report[18]. Ancestral/conserved clade is most conserved to KAI2 phylogenetically but nonresponsive to karrikin and SL; diverse/fastest-evolving clade is corresponding to D14 being responsive to SL but not karrikin; while intermediate clade is responsive to karrikin but not SL.

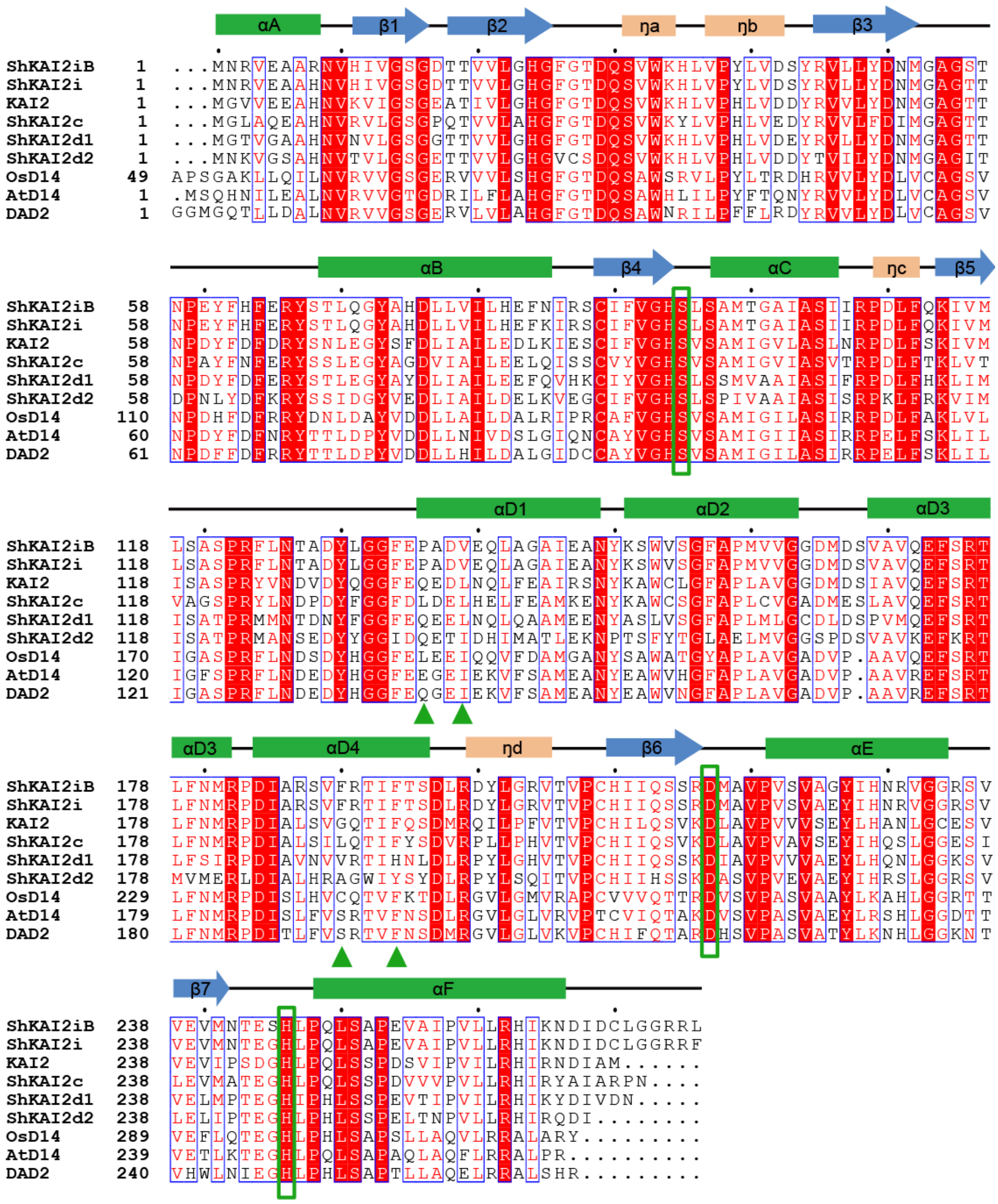

Supplementary Figure 2. Multiple sequence alignment of KAI2/D14 proteins. Identical residues are shaded red, and homologous residues are shown as red letters. Green boxes represent catalytic triad residues. The secondary structure element of ShKAI2iB is shown at the top of the alignment (α, α-helix; η, 3_{10}-helix; and β, β-strand). Non-conserved residues (Val139, Pro136, and Phe190) of ShKAI2iB are highlighted with green triangles.

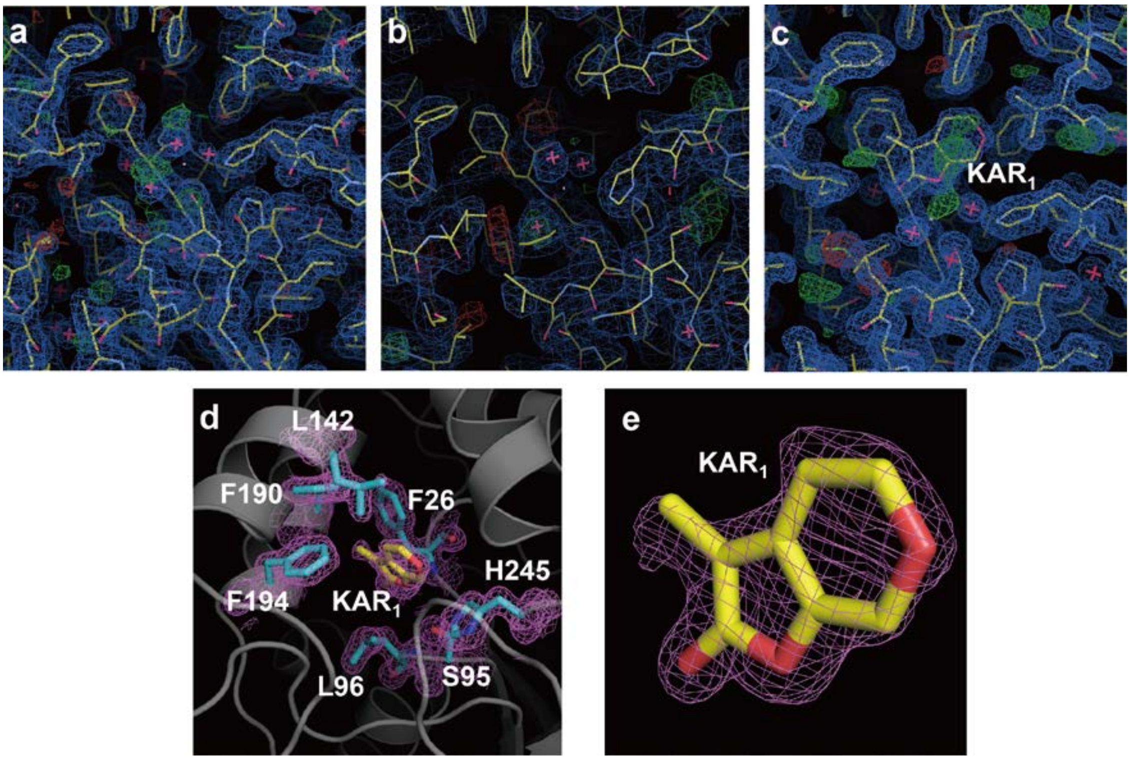

Supplementary Figure 3. Electron density maps of ShKAI2iB and its complex with KAR_1. (**a**), (**b**), (**c**), Electron density maps for the ligand-binding cavity of apo-ShKAI2iB (**a**), ShKAI2iB-intermediate (**b**) and ShKAI2iB-KAR_1 complex (**c**) are shown. The mesh diagram represents F_o-F_c (green, +; red, –) at contour level of 3.0σ and 2F_o-Fc (blue) maps at contour level of 1.0σ. (**d**) 2F_o-F_c map (1.0σ) around KAR_1 for the ShKAI2iB-KAR_1 complex structure. (**e**) The mesh diagram represents F_o-F_c omit map of KAR_1 contoured at level of 3.0σ from ShKAI2iB-KAR_1 complex structure.

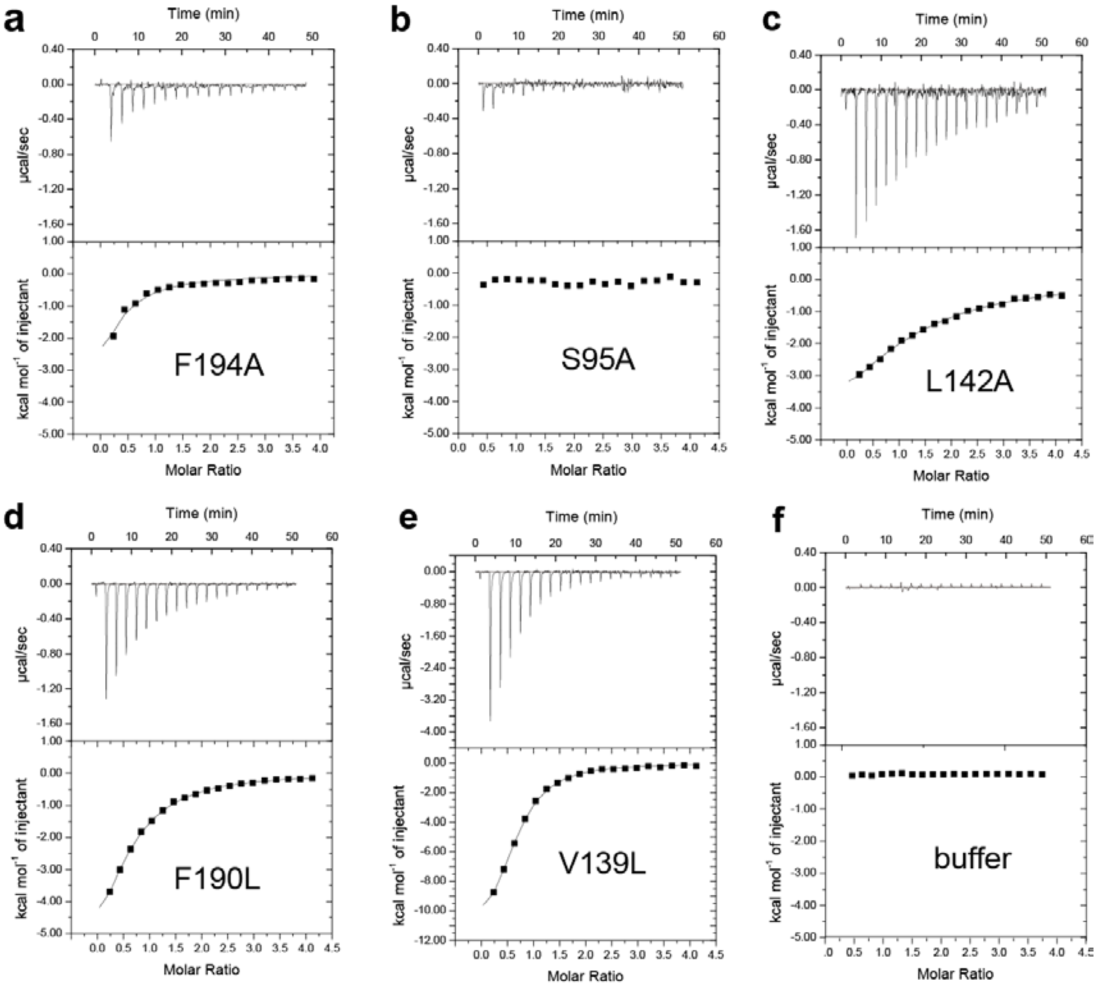

Supplementary Figure 4. Results of ITC experiments between ShKAI2iB mutants and KAR_1. ITC experiments were performed by titrating KAR_1 into ShKAI2iB mutants (**a**, F194A; **b**, S95A; **c**, L142A; **d**, F190L; **e**, V139L) or into buffer as a negative control (**f**). The upper panel illustrates raw ITC data. The bottom panel represents data of the enthalpy change derived from the upper panel. Corresponding thermodynamic results are shown in Table S2.

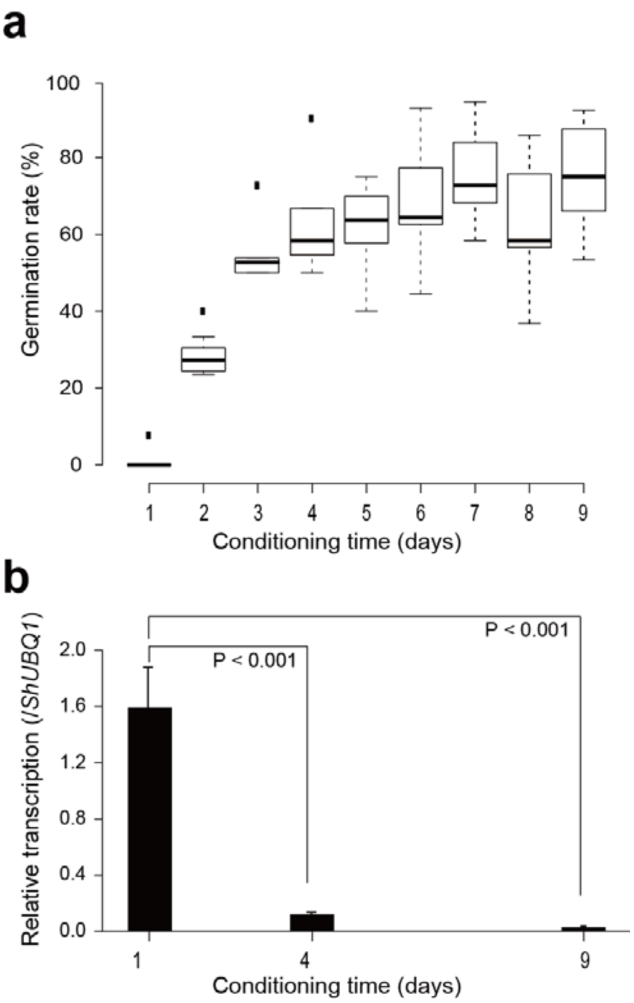

Supplementary Figure 5. Transcript level of *ShKAI2iB* during *Striga* seed conditioning. The *Striga* germination rate and the ShKAI2iB gene expression during the seed conditioning period. (**a**) Changes in the *Striga* seed germination rate during the seed conditioning period. *Striga* seeds were treated with 0.1 μM of GR24. After further incubation at 30°C for 3 days, GR24-treated seeds were microscopically evaluated for germination. Center lines show the medians; box limits indicate the 25th and 75th percentiles as determined by R software; whiskers extend 1.5 times the interquartile range from the 25th and 75th percentiles, outliers are represented by dots. *n* = 5, 7, 5, 5, 7, 7, 7, 7, 7 sample points. (**b**) Relative transcript levels of *ShKAI2iB* in *Striga* seeds conditioned for indicated days. The data means ± SE of three samples. Student's t test was used to determine the significance of differences relative to the transcript level in *Striga* seeds conditioned for 1 day.

Supplementary Table 1. Statistics of X-ray data collection and refinement.

	**ShKAI2iB**	**ShKAI2iB-I**	**KAR_1-bound ShKAI2iB**
**Data collection**			
Beamline	R-AXIS VII	PF AR-NW12A	PF AR-NW12A
Wavelength (Å)	1.5418	1.0000	1.0000
Space group	$P6_122$	$P6_122$	$P6_122$
Unit-cell parameters (Å)	75.9, 75.9, 181.5	76.0, 76.0, 180.0	75.8, 75.8, 181.3
Resolution range (Å)	19.7−2.0	24.9−2.6	19.7−1.2
No. of unique reflections	21039 (1469)	9559 (475)	96642 (4707)
Redundancy	18.7 (9.6)	20.2 (20.9)	20.6 (3.6)
Completeness (%)	99.6 (96.5)	100.0 (99.9)	99.9 (99.6)
R_{sym}	0.063 (0.370)	0.102 (0.626)	0.065 (0.390)
$\langle I/\sigma(I) \rangle$	36.2 (6.1)	25.0 (5.9)	97.8 (5.3)
**Refinement**			
No. reflections	19959	9057	91454
R_{factor}/R_{free} (%)	18.0/22.5	20.7/26.6	14.1/15.5
No. atoms			
Protein	2142	2103	2102
Ligand	-	-	11
Water	236	41	209
B-factors (Å^2)			
Protein	29.8	65.3	21.9
Ligand	-	-	21.9
Water	38.3	47.0	31.4
R.m.s. deviations			
Bond lengths (Å)	0.009	0.008	0.011
Bond angles (º)	1.310	1.093	1.694

*Values in parentheses are for the highest-resolution shell.

Supplementary Table 2. Thermodynamic parameters determined by ITC.

Mutant	K_D (μM)	ΔH (kcal mol^{-1})	ΔS (cal mol^{-1} deg^{-1})	ΔG (kcal/mol)
WT	77.6 ± 3.4	–6.48 ± 0.44	–3.99	–5.35
V139L	35.6 ± 7.0	–12.87 ± 0.03	–24.8	–6.96
L142A	196 ± 24	–7.00 ± 0.70	–7.90	–4.76
F190L	187 ± 43	–11.71 ± 8.89	–24.6	–4.75
F194A	107 ± 10	–13.24 ± 63.94	–28.7	–5.11
